# The Definition of Placebo in the Informed Consent Forms of Clinical Trials

**DOI:** 10.1371/journal.pone.0113654

**Published:** 2014-11-25

**Authors:** Astrid Hernández, Josep-E. Baños, Cristina Llop, Magí Farré

**Affiliations:** 1 Human Pharmacology Unit, Hospital del Mar Medical Research Institute-IMIM, Barcelona, Spain; 2 Department of Pharmacology, Therapeutics and Toxicology, Universitat Autònoma de Barcelona, Bellaterra, Spain; 3 Department of Experimental and Health Sciences, Universitat Pompeu Fabra, Barcelona, Spain; Universidad de Granada, Spain

## Abstract

**Aim:**

Lack of knowledge concerning the nature of placebo and why it is necessary may influence the participation of patients in clinical trials. The objective of the present study is to review how placebo is described in written information for participants in clinical trials to be evaluated by a Human Research Ethics Committee.

**Methods:**

All research protocols submitted for evaluation in a Spanish hospital during 2007–2013 were reviewed. The main characteristics of the studies using a placebo were collected. Three authors read each of them to determine how the term “placebo” was explained and if there was any comment on its efficacy and safety.

**Results:**

Two thousand seven-hundred and forty research protocols were evaluated, of which three hundred and fifty-nine used a placebo. Pharmaceutical companies sponsored most placebo-controlled clinical trials (91.9%), and phase III studies were the commonest (59.9%). Oncology (15.0%), cardiology (14.2%), and neurology (13.1%) made the greatest contributions. A review of the informed consent forms showed that placebo was described in a similar manner in most studies: the explanation was limited to between four and eight words. Very few gave information about the risks of its use or adverse reactions from its administration. None of the studies provided details about the placebo effect. And 23 lacked any information about placebo at all.

**Conclusions:**

Explanations about placebo in informed consent forms is often scarce, and information about the placebo effect and associated risks are absent. This situation may influence a full understanding of placebo by participants in clinical trials and might reduce their informed decision to participate.

## Introduction

Placebos, and the corresponding placebo effect, have a long tradition in medical practice: physicians have used them, consciously or not, since ancient times to treat patients [1]. Currently, a number of surveys have shown that the use of placebo is widespread in clinical practice. For example, only 28% of Swiss primary care providers reported that they never used placebo interventions [2]. And in Israel, 60% of physicians and nurses admitted to employing placebos, the majority reporting them to be an effective therapeutic alternative [3]. In agreement with these results, patient attitudes to placebos were somewhat similar. A survey in New Zealand of primary-care patients described that they accepted a placebo in certain clinical situations: when it was for the benefit of the patient, at the patient's request, or when there appeared to be no other treatment available [4].

In recent years, evidence has accumulated to enhance the understanding of the physiological processes that contribute to the placebo effect [5]. However, its role in clinical research has always been a controversial issue, mainly for ethical reasons. Those who are against a placebo in clinical trials consider that its administration is unfair because, according to the Declaration of Helsinki, physicians should employ the best clinical treatment in any setting [6]. Nevertheless, several reasons have been put forward to justify the use of a placebo in clinical research as a methodological tool that permits more reliable results [7]. This approach seems to be ethically acceptable, provided that it is supported by justified methodological reasons and it does not expose patients to deleterious effects that may follow the its administration (for instance, prolonged pain in the case of analgesic trials), and that it has adequate consideration in the study protocol (for example, the possibility of rescue treatment). In this respect, the World Medical Association Declaration of Helsinki (2013) states that “where no proven intervention exists, the use of placebo, or no intervention is acceptable” [8].

Clinical trials have been the cornerstone of medical research since the Medical Research Council trial of streptomycin for tuberculosis was published in 1948 [9]. In the following years, the methodology has been refined to avoid any bias in the design, management, and interpretation of clinical trials. Placebo has been one of the most relevant factors introduced to permit these improvements [10]. In clinical research, ethical codes and legal regulations establish the need for patients to receive adequate information about the characteristics of the trial in which they will be enrolled. To meet these requirements, informed consent forms (ICFs), compulsory before patients are included in any clinical trial, are proof that this information has been provided [11].

Willingness to participate in clinical trials that use a placebo may be influenced by the severity of the disease. For example, 70% of patients with cancer would decline to participate in a study with new drugs due to fears of receiving placebo, or the belief that the standard therapy is better than the experimental treatment, even though placebo is rarely used in cancer trials [12]. In a hypertension trial, 24% of the patients were concerned about the possibility of receiving a placebo. Whilst the proportion of subjects who might receive placebo influenced patient enrolment decisions it was not a key determinant of recruitment efficiency [13]. It has been observed that the most common concerns that could hinder participation are: cessation of current medication (56%), inconvenience/annoyance (38%), fear of known side-effects (35%), and the possibility of receiving placebo (24%) [14]. From the perspective of the researchers it has been reported that they conceded less importance to methodological issues, such as placebo administration, in the explanation of clinical trials to participants [11].

The reasons summarized above suggest that a misunderstanding of what a placebo is, and the reason for its use, may impede the participation of patients in studies that employ it as a comparison treatment. Systematic analyses of how placebo is explained in information leaflets are scarce: we only found one in the literature [15]. The authors focused their study on comparing the information of placebo with target treatments from a major registry of current United Kingdom clinical trials. They concluded that the definition of placebo was incomplete and often inaccurate. They recommended improving such information to avoid the jeopardy of informed consent. A comprehensive study evaluating the details provided to patients participating in clinical trials is, therefore, clearly justified. The objective of the present study is to review how placebo was described in the ICFs of clinical trial protocols to be evaluated by a Human Research Ethics Committee over the previous six years.

## Methods

All the research protocols submitted for evaluation to the Human Research Ethics Committee of *Parc de Salut Mar* (CEIC-PSMAR) in the period 2007–2013 were reviewed. *Parc de Salut Mar* is the organization responsible for the management of several public health centres in Barcelona, Spain, and includes a number of hospitals, primary-care centres, nursing homes, and mental-health units. The great majority of protocols that included placebo were done in the facilities of Hospital del Mar (354/359), four in a mental health centre (Centre Dr. Emili Mira) and one exclusively in the Hospital de la Esperanza (Department of Radiotherapy).

### Definitions

For operational purposes we defined placebo as “An inert substance usually prepared to look as similar to the active product investigated in a study as possible” [16], and Placebo effect as “A nonspecific term used to encompass any (usually beneficial) changes that occur within a group ‘treated’ with placebo” [16].

### Study procedure

The following steps were carried out:

- Preparation of an MS Excel spreadsheet to collect data from the studies and facilitate the quantitative content analysis.
- Identification of the protocols that included placebo as a control treatment. The summaries of the research protocols were read by three of the authors. In the case of disagreement the protocol was again reviewed, and a final decision made by consensus from the three evaluators.
- Collection of the main characteristics of each clinical trial using a placebo (aim, design, objective, disease or medical condition, phase of clinical trial, name and type of sponsor, medical specialty of the principal researcher).
- Analysis of the placebo definition. Three of the authors separately reviewed how placebo was explained in each ICF and annotated its exact description in the spreadsheet. Attention was focused on how placebo was defined with regard to its appearance and pharmacological effect, as well as any reference to its efficacy and safety. Aspects concerning the use of placebo in the randomization process were not considered in the current study.
- Coding of the placebo definition. The authors reviewed the contents of the spreadsheet to code the definitions of placebo. It was agreed that the information should be categorized into four groups according to their key characteristics: no definition, definition based only on appearance, definition based on pharmacological effect, or definitions that include both. Two researchers assigned the definitions. The inter-rater agreement was calculated as the percentage of same code assignation by both researchers [17]. In the case of agreement not being reached, the corresponding author assigned the final code for definition.
- Translation of the placebo definition. All the ICFs were written in Spanish. For the purpose of the present article, some of the definitions were translated to English and then back-translated to assure that the meaning was retained during the process [18]. The sentences obtained at the end of this process appear in Table 1.

**Table 1:** Most frequent explanations of what placebo is on the patients' information sheets (n = 359).

Placebo description	n (%)
**Not described**	**23 (6.4)**
**Appearance**	**25 (6.9)**
“Same/similar look”	12 (3.3)
“Pill/tablet of sugar”	9 (2.5)
“Saline solution”	4 (1.1)
**Effect**	**121 (33.8)**
“A substance/capsule without drug or active medicine”	55 (15.4)
“A substance/capsule/drug without pharmacological activity”	26 (7.2)
“A substance/capsule/drug without activity”	29 (8.1)
“A substance/capsule/drug with no effect”	11 (3.1)
**Appearance and effect**	**190 (52.9)**
“Same look, without pharmacological or therapeutic activity”	13 (3.6)
“Same or similar look, without any active ingredient or drug”	152 (42.3)
“Same look, inactive drug without any effect”	25 (7.0)
**Total**	**359 (100)**

Descriptive analysis was carried out using SPSS 12.0.

### Ethics Statement

The study was approved by the Local Research Ethics Committee (CEIC-PSMAR, Number 2011/4234).

## Results

A total of 2740 research protocols were evaluated in this survey from January 2007 to December 2013. Three hundred and fifty-nine (13.10%) of them had used placebo in therapeutic or preventive trials, mainly as a control (n = 167, 46.5%) or add-on treatment (n = 129, 35.9%), but also in a double-dummy design (n = 63, 17.5%). All informed consent forms were in a written format. Pharmaceutical companies sponsored most placebo-controlled clinical trials (n = 330, 91.9%), the remaining studies came from independent researchers (n = 29, 8.1%). Phase III studies were the commonest (n = 215, 59.9%), followed by Phase II (n = 93, 25.9%), Phase IV (n = 38, 10.6%), and Phase I (n = 13, 3.6%). Most of the Phase II, III and III studies were multicentre and multinational clinical trials (94%). Analysis by medical specialties showed that clinical trials from oncology (n = 54, 15.0%), cardiology (n = 51, 14.2%), and neurology (n = 47, 13.1%) made the greatest contributions to the total number of studies (Table 2). We did not find differences in placebo descriptions or number of words used when analyzing the study protocols by Phase of the trial, medical specialty or center of reference.

**Table 2:** The commonest medical specialties contributing to the total number of studies including placebo (they represent 72.8% of the total number of studies including placebo).

Medical specialty	n (%)
Oncology	54 (15.0)
Cardiology	51 (14.2)
Neurology	47 (13.1)
Digestive	27 (7.5)
Pulmonology	22 (6.1)
Rheumatology	22 (6.1)
Nephrology	20 (5.6)
Dermatology	12 (3.3)
Psychiatry	12 (3.3)
Clinical Pharmacology	10 (2.8)
Infectious Diseases	10 (2.8)
**Total**	287 (79.8)
**Others**	72 (20.2)
	359 (100)

The review of the ICFs showed that the definition of placebo was explained in a similar manner in most of the studies. The explanations concerning placebo were classified according to its main description: its appearance (n = 25, 6.9%), effects (n = 121, 33.8%), or both (n = 190, 52.9%). The majority of the clarifications about placebo in the ICFs referred simultaneously to its appearance and effects. The inter-rater agreement was 94% (336/359). The most common explanations are summarized in Table 1. Only twenty-three (6.4%) ICFs lacked any description about the use of placebo. In the majority of the ICFs, placebo was clarified although no explanation about its risks, such as patient deterioration as a consequence of the delay of an effective treatment, was given. No information was found in any study about the placebo effect or the adverse reactions that could ensue from the administration of placebo (i.e. the nocebo effect). In the forms in Spanish, the mean number of words used to define placebo was 14, whereas in the translated English version it was 12.

## Discussion

Our most relevant findings are that in the ICF placebo is generally described in an unsatisfactory manner and with no reference to its possible advantages or disadvantages. We consider that an explanation of only four to eight words, without any information about its possible benefits-harm (the placebo effect) or the implications of being treated with it (the nocebo effect and the risks of its use), is insufficient to understand the meaning of placebo. Nevertheless, this short description is similar to that recommended in most examples of ICFs found on the IRB web pages in the majority of hospitals and research centres, and those of the National Institutes of Health-NIH in the United States of America (see Table 3 for some examples). In the paper of Bishop et al [15], a similar result was found in 45 participant information leaflets. The explanation of placebo was just as limited as in our findings and only in one leaflet was placebo described as capable of eliciting effects. This is an important point, since patients need to know what receiving placebo as a therapy during the trial specifically means. A better explanation is clearly needed to ensure the full understanding of the process of the clinical trial when patients are invited to participate. Our findings confirm the assumption that the improvement of the quality of informed consent is a pending issue. In this respect, we agree with Resnick [14] when he writes, “While it is important to conceive of informed consent as a process, let's not forget the consent document. It may only be words written on the printed page, but those words matter a great deal. Informed consent documents should be readable, accurate and thorough”. We believe that this statement also applies to the correct description of placebo.

**Table 3:** Descriptions of placebo found on the selected webpages of some organizations.

Organization	Webpages	Descriptions of placebo
ClinicalTrials.gov	http://clinicaltrials.gov/ct2/about-studies/glossary#P	A substance that does not contain active ingredients and is made to be physically indistinguishable (that is, it looks and tastes identical) from the actual drug being studied.
ICH guidelines: E10: Choice of Control Group and Related Issues in Clinical Trials (Section 2.1)	http://www.ich.org/fileadmin/Public_Web_Site/ICH_Products/Guidelines/Efficacy/E10/Step4/E10_Guideline.pdf	A "dummy" treatment that appears as identical as possible to the test treatment with respect to physical characteristics such as colour, weight, taste and smell, but that does not contain the test drug.
EU Clinical Trials Register	https://www.clinicaltrialsregister.eu/doc/EU_Clinical_Trials_Register_Glossary.pdf	A placebo is a control substance (a dummy treatment) that is given to people taking part in a clinical trial.
Medline Plus Medical Library	http://www.merriam-webster.com/medlineplus/placebo	1: A usually pharmacologically inert preparation prescribed more for the mental relief of the patient than for its actual effect on a disorder. 2: An inert or innocuous substance used especially in controlled experiments testing the efficacy of another substance, such as a drug.
National Institutes of Health (NIH)	http://www.nih.gov/health/clinicaltrials/glossary.htm	A placebo is a pill or liquid that looks like the new treatment but does not have any treatment value from active ingredients.
NIH-Clinical Center	http://www.cc.nih.gov/participate/faqaboutcs.shtml	Placebos are harmless, inactive substances made to look like the real medicine used in the clinical trial.
NIH-National Heart lung and Blood Institute (NHLBI-NIH)	http://www.nhlbi.nih.gov/childrenandclinicalstudies/terms.php#Placebo	Placebo is a pill, liquid or powder that has no active medicine in it. It's a fake.
National Library of Medicine – Medical Subject Headings (MeSH)	http://www.nlm.nih.gov/cgi/mesh/2014/MB_cgi?mode=&index=10448&field=all&HM=&II=&PA=&form=&input=	Any dummy medication or treatment.
World Health Organization (WHO)	http://whqlibdoc.who.int/publications/2009/9789241547727_eng.pdf	In the context of research, a placebo is a substance or procedure which patients accept as a medicine or therapy, but which actually has no specific therapeutic activity for their conditions.

Few studies analyse how the concept of placebo, as well as the implications of its use, is explained in the information leaflets that are given to patients when they are asked to participate in clinical trials. Our literature search only provided us with the study of Bishop et al [15]. The authors focused their research on comparing the information on placebo and target treatments, and they concluded that the explanation about placebo was incomplete and often inaccurate. As the authors stated, their study had the limitation of a low response rate from the named contact personnel (13.5%), and only one of the studies which were analysed was commercial. They recommended improving such information to avoid the jeopardy of informed consent. Our study analysed a larger number of trials and only focused on information concerning the placebo. Most of the clinical trials which we analysed were sponsored by pharmaceutical companies. This provides a very different picture to that of Bishop et al [15]. As our sample was obtained from the whole population of study protocols reviewed by the Human Research Ethics Committee, our work gives a wider view of the studies currently being carried out in European countries. Another difference is that a large proportion of the studies analysed by Bishop et al [15] were phase IV trials, whereas our sample included mainly phase III trials (60%). We believe, therefore, that our findings strengthen the previous available evidence which suggests that information on placebo in informed consent documents is unsatisfactory [15].

Controversy still persists over the actual understanding that participants have after reading the information sheet of clinical trials [15]. Several authors have reported low levels of comprehension in relation to the process of informed consent [19], [20]–[26] and shortfalls have been confirmed [30]–[34]

Even if this is the case, to our knowledge patients are willing to participate in clinical trials that use placebos and accept their use for clinical research [27]–[29]. Several studies have confirmed shortfalls in understanding [30]–[34]. Such a situation reinforces the need for studies to evaluate which parts of an ICF are badly understood in order to take adequate measures [35]. If not, patients will give their consent but not their informed consent, which clearly violates the autonomy principle. Even worse, participants in the study may not understand why they receive placebo (i.e. non-expected benefit) instead of the tested drug (i.e. expected benefit). It has been reported that there is no correlation between the amount of information given in an ICF and the patient's decision to participate [29]. In patients of a Phase III clinical trial the improvement in ICF readability did not increase comprehension [36], possibly due to the fact that up to 69% of participants sign without reading [37]. Empirical research has shown that patients are able to understand and use only a portion of the information provided by consent forms [38], [39].

The information provided to the patients, with respect to placebo issues, is sometimes limited by the fear that detailed aspects of the risks and benefits of placebo may hinder acceptance to participate [40]. However, from an ethical point of view, adequate information on placebo is the best way to ensure that participants will make informed choices about their participation, as has been suggested by the random-allocation concept [41]. While patients appear to be willing to be included in clinical trials with placebo [27]–[29], some empirical evidence suggests the existence of a placebo group may limit patient participation. In a clinical trial of hormone-replacement therapy, 30% of the women reported they were prepared to participate in a study with a placebo arm, whereas 39% would do so without such an arm [42]. Although these differences are not statistically significant, they do indicate a trend to reduce willingness to participate, at least in preventive trials. Golomb et al. [43] have reported that the composition of placebo is rarely disclosed in published clinical trials, and it is possible that the same happens in information sheets. This fact might increase reluctance to receive a completely unknown treatment that may actually improve a significant fraction of patients' health status [44]–[46].

A possible limitation of our findings is the narrow definition of placebo. Other authors, such as Bishop et al. [15], have considered more information in their analysis, for instance the purpose of implementing placebo and the probability of receiving it. We consider, however, that these considerations are more related with the design of the clinical trial and not the strict definition of placebo. A specific study analysing data about comparative treatments and/or the randomization process may be more useful for this objective.

Another important point is the fact that our study has been carried out in one centre of one country; a critical issue when external validity of the results is considered. We would like to emphasize that most of the clinical trials in our centre that include placebo were multicentre-multinational Phase II–III studies sponsored by multinational pharmaceutical companies. With the exception of language, the protocol and ICF were the same for all of the countries involved (mostly from Europe and the USA). However, further research is needed in such other countries to test the generalizability of our results.

Our results indicate that information about placebo in informed consent forms is often scarce, and the explanations about placebo effect and associated risks are practically absent. Its influence on the willingness of subjects to participate in clinical trials is unknown and should be studied in the future. To ensure a truly informed consent, participants must be knowledgeable about what placebo means. Patients should be told what placebo really is and why they might be receiving this option during the clinical trial. For this reason, we suggest that an explanation like placebo is a substance without any biological action that we use to ascertain the actual efficacy of a drug, as we know that expectations of patients and physicians on the effect of treatment may change the final effect of any drug.
